# Immune Reconstitution after Haploidentical Donor and Umbilical Cord Blood Allogeneic Hematopoietic Cell Transplantation

**DOI:** 10.3390/life11020102

**Published:** 2021-01-29

**Authors:** Hany Elmariah, Claudio G. Brunstein, Nelli Bejanyan

**Affiliations:** 1 Department of Blood and Marrow Transplant and Cellular Immunotherapy, Moffitt Cancer Center, Tampa, FL 33612, USA; nelli.bejanyan@moffitt.org; 2 Division of Hematology, Oncology, and Transplantation, University of Minnesota, Minneapolis, MN 55455, USA; bruns072@umn.edu

**Keywords:** allogeneic transplant, immune reconstitution, haploidentical, umbilical cord blood

## Abstract

Allogeneic hematopoietic cell transplantation (HCT) is the only potentially curative therapy for a variety of hematologic diseases. However, this therapeutic platform is limited by an initial period when patients are profoundly immunocompromised. There is gradual immune recovery over time, that varies by transplant platform. Here, we review immune reconstitution after allogeneic HCT with a specific focus on two alternative donor platforms that have dramatically improved access to allogeneic HCT for patients who lack an HLA-matched related or unrelated donor: haploidentical and umbilical cord blood HCT. Despite challenges, interventions are available to mitigate the risks during the immunocompromised period including antimicrobial prophylaxis, modified immune suppression strategies, graft manipulation, and emerging adoptive cell therapies. Such interventions can improve the potential for long-term overall survival after allogeneic HCT.

## 1. Introduction

Allogeneic hematopoietic cell transplantation (HCT) offers the only potential cure for many high-risk hematologic malignancies. The therapeutic benefit of allogeneic HCT is, in part, due to an alloreactive graft-versus-tumor (GVT) response whereby the donor immune system recognizes the recipient tumor cells as foreign and eradicates them [1]. However, this same alloreactivity can also lead to toxicity such as graft-versus-host disease (GVHD) where the donor immune system attacks the recipient, or graft rejection where the recipient immune system attacks the donor cells [2,3]. Control of these bidirectional immune responses requires modulation of the lymphodepleting conditioning regimen and immune suppressive therapies (IST) which decrease the risks of graft rejection and GVHD, but also increase risks of infection [4,5,6,7]. Antimicrobial prophylaxis and vaccination, thus, also become critical elements of successful HCT [8].

In addition to IST, selection of a human leukocyte antigen (HLA) matched donor has long been considered a critical consideration to mitigate the risks of toxic alloreactivity and consequent transplant related mortality (TRM) [9,10]. Historically, HLA matched sibling donors (MSD) have been the preferred donor option followed by HLA matched unrelated donors (MUD). However, due to the Mendelian inheritance pattern of HLA haplotypes, the likelihood of a patient’s each sibling being a full HLA match is 25% and the likelihood of identifying a MUD varies from 10–80% depending on the ethnic and racial background of the patient [11]. To improve access to HCT for patients lacking an HLA matched donor, alternative donor platforms such as haploidentical (haplo) related donor HCT and umbilical cord blood transplant (UCBT) have been developed [12]. Specifically, haplo HCT with use of posttransplant cyclophosphamide (PTCy) has emerged as a favorable strategy that yields rates of GVHD, TRM, and overall survival (OS) that are nearly equivalent to matched donor HCT [13,14]. Further, in a recent Blood and Marrow Transplant Clinical Trials Network (BMT CTN) phase III trial, haplo HCT with PTCy was well tolerated and resulted in better overall survival (OS) compared to UCBT [15]. Nonetheless, both approaches are considered appropriate alternative donor HCT strategies and the choice of haplo versus UCBT is still largely dependent on institutional expertise [13].

The feasibility of modern approaches to HCT, including expansion of the donor pool to haplo and UCBT, requires unique manipulations to the immune systems of both donor and recipient cells to allow for successful donor engraftment and prevention of GVHD. However, these manipulations can result in profound effects on immunity that impact rates of infection and relapse of the primary malignancy [13,15]. Here, we describe the immune reconstitution after allogeneic HCT in general, special considerations for haplo HCT and UCBT, as well as resultant impacts on clinical outcomes and considerations for management, particularly in the context of hematologic malignancies.

## 2. Kinetics of Immune Reconstitution after Allogeneic HCT

In practice, allogeneic HCT consists of a conditioning regimen (chemotherapy and/or total body irradiation (TBI)) to suppress recipient alloreactivity against the donor and allow engraftment of the donor hematopoietic stem cells, followed by infusion of the donor hematopoietic stem cells and subsequent initiation of IST. This procedure acutely results in severe immune compromise followed by gradual immune reconstitution [16]. While immune reconstitution varies depending on the specific transplant platform, there are uniform patterns that inform the likelihood of specific immunologic complications over time.

### 2.1. Early Immune Reconstitution

The conditioning regimen given prior to transplant results in profound cytopenias that nadir in the week following stem cell infusion. This results in depletion of both innate and adaptive immunity. During this period, susceptibility is high to bacterial and fungal infections so antibacterial and antifungal prophylaxis is standard [8]. In general, the innate immune system begins to recover first. Monocyte engraftment begins in the two weeks after transplant followed closely by neutrophil recovery [17]. Concurrently, non-hematopoietic innate immunity, such as mucosal barriers, heal from injury caused by the conditioning regimen [17]. At most centers, the resolution of neutropenia and mucosal injury are key landmarks required for hospital discharge and discontinuation of antibacterial and antifungal prophylaxis. In the weeks following neutrophil engraftment, natural killer (NK) cells recover [18,19,20,21,22,23]. These cells are increasingly recognized as essential to the GVT effect that prevents disease relapse [20,21,22,23].

### 2.2. Late Immune Reconstitution

Though the innate immune system quantitatively recovers in the first weeks after HCT, these cells may not be functionally competent. Indeed, functional recovery of the hematopoietic innate immune system typically occurs in the 4–12 months after transplant [17,24,25]. The adaptive immune system, including the cellular immune response and humoral immune response, requires functional T-lymphocytes and B-lymphocytes. These cells begin recovering in the months after transplant but may require years to reach full competence [26,27,28].

The recovery of the cellular immune response, of particular importance for immunity against viral pathogens and graft-versus-tumor, occurs in two phases. First, immunocompetent T-cells in the donor graft may undergo clonal expansion [16,29]. Second, naïve T-cells from the donor may be expanded in the thymus of the recipient [16,29]. The humoral immune response resulting in adequate antibody response, requires recovery of T-cells as well as functional B-cells, which recover between 3 months–1 year after transplant [17,30]. It is during this phase of recovery that post-HCT vaccinations are typically initiated [8].

### 2.3. Factors Influencing Immune Reconstitution

The expected immune reconstitution described is general and may vary considerably between individual patients due to both modifiable and fixed aspects of HCT [17]. First, advanced age of either the donor or the recipient can result in slower immune recovery due slower engraftment with aging marrow [31,32]. Further, T-cell immune reconstitution requires a functional thymus and, thus, may be significantly limited in older patients who can have thymic atrophy [29]. HLA matched donors have also been shown to yield better immune reconstitution, possibly because HLA mismatched may lead to more mixed lymphocyte reactions and host-versus-graft alloreactivity that can delay immune reconstitution [25,33,34]. More intensive conditioning regimens can result in more rapid engraftment, though may also slow early lymphocyte recovery [35]. Graft related factors may also contribute to immunity, with peripheral blood stem cells grafts resulting in more rapid engraftment than marrow grafts [17,36]. Grafts with higher stem cell dose or higher T-cell content also may engraft more quickly, resulting in more rapid immune reconstitution [37,38,39]. Conversely, T-cell depletion of the graft, often used for GVHD prevention, results in higher rates of graft rejection as well as slower immune reconstitution and increased risk of infections [40,41]. Finally, the GVHD prophylactic regimen, its duration of administration, and onset of acute or chronic GVHD are all associated with impaired immune reconstitution [3,8,42].

### 2.4. Clinical Significance of Immune Reconstitution

The kinetics of immune reconstitution correlate temporally with expected transplant related complications. In the period preceding donor cell engraftment, the marrow is aplastic. The resultant profound neutropenia leads to a period of high risk for bacterial and fungal infections, generally in the first 30 days after HCT [43,44]. Between days 30–100 after HCT, as cell mediated immunity slowly recovers, the highest risk infections shift towards viral reactivation such as cytomegalovirus (CMV), human herpesvirus 6 (HHV–6), or Epstein–Barr virus (EBV), in addition to pneumocystis pneumonia (PCP) [8,45,46]. During this period, acute GVHD, a T-cell mediated process, also emerges, occurring in ~20–50% of HCT patients and can result in skin rashes, gastrointestinal toxicity, hepatic injury, infections, and mortality [4,5,47].

Beyond day 100, though infectious immunity steadily improves, chronic GVHD will occur in up to 60% of HCT patients, though may be lower with modern approaches even with a haplo, and cord blood transplant [3,4,5,15,47,48]. The occurrence of chronic GVHD is a risk factor for subsequent infections, due to both inherent immune dysregulation as well as increases in IST to control chronic GVHD [49].

In addition to transplant related toxicity, post-HCT immune reconstitution is necessary to prevent relapse and cure the underlying hematologic malignancy through the immunologic GVT effect. The GVT effect became clinically apparent in studies showing greater HLA disparity resulted in reduced risks of relapse, while genetically similar identical twin donors result in higher risks of relapse [1]. It is now understood that cytotoxic T-cells are critical to the GVT effect, eliminating tumor cells through secretion of granzyme B as well as apoptosis via FAS ligands [50]. The significance of T-cells to the GVT effect has been demonstrated clinically as donor lymphocyte infusions are able to eradicate active tumor, while T-cell depletion results in higher risks of relapse [40,51]. The GVT effect can be triggered by HLA mismatch, as well as host minor histocompatibility antigens and tumor related neoantigens [52]. Thus, the ability of the cytotoxic T-cells to distinguish healthy host tissue from tumor cells is limited, and GVT and GVHD often overlap.

In recent years, there has been increasing focus on the role of NK cells as mediators for the GVT effect [53]. NK cell function is dependent upon receptor/ligand interactions resulting in activating signals or inhibitory signals [54,55,56]. The balance of activation and inhibition leads to either cell killing or tolerance [55,56]. Interactions between the NK cell killer immunoglobulin-like receptor (KIR) with self HLA class I molecules, which are expressed uniformly on healthy host tissue, lead to inactivation [20,21,57,58]. In the setting of allogeneic HCT, certain combinations of donor activating KIR types and recipient HLA subtypes promote NK cell activation, leading to a more potent GVT effect without an increase in GVHD [53,54,59,60,61,62,63,64]. An ongoing prospective, multicenter trial is evaluating the utility of incorporating donor KIR type in selection of allogeneic HCT donors for patients with acute myeloid leukemia (NCT02450708). Further, the ability of tumor cells to down regulate HLA class I creates a mechanism by which NK cells can differentiate between tumor and healthy host tissue, thus leading to activation and tumor killing [65,66,67].

## 3. Haploidentical Donor Transplant

Because of the inheritance patterns of HLA haplotypes, parents and children will be HLA haploidentical matches and siblings have a 50% likelihood of being haploidentical matches. As a result, patients in need of transplant have a >90% likelihood of having a suitable HLA haploidentical related donor [68]. However, the significant HLA mismatch between the haplo donor and the recipient results in intense bidirectional alloreactivity whereby the donor immune system attacks the recipient (GVHD) and the recipient immune system attacks the donor cells (graft rejection) [69]. Early studies of haplo transplant, thus, resulted in unacceptable toxicity that precluded the use of this strategy for many years [69].

### 3.1. Approaches to Haploidentical Transplant

The acute alloreactivity that occurs after haplo HCT is mediated primarily by donor and recipient T-lymphocytes. Multiple regimens have been developed to target T-cell function to mitigate toxicity. Three strategies have become the most utilized: (1) high-dose PTCy; (2) ex vivo T-cell depletion (TCD) with “megadose” CD34+ cells; and (3) the ”GIAC” regimen (GCSF-stimulation of the donor; intensified immunosuppression through post–transplantation CsA, mycophenolate mofetil (MMF), and short-course methotrexate; antithymocyte globulin (ATG) added to conditioning to help prevent GVHD and aid engraftment; and combination of PBSC and bone-marrow allografts) [14,70,71].

### 3.2. Immune Reconstituion after Haploidentical Transplant

Because of the higher risks of GVHD with HLA mismatched donors, the GVHD prophylaxis regimens used for haplo donor HCT are more immune suppressive than those used in matched donor HCT. Haplo HCT with PTCy has emerged as the haplo platform of choice in the United States. Immune reconstitution with this regimen has been compared retrospectively to matched donor and UCBT [72]. Compared to the MSD group, the haplo with PTCy group had a higher risk of CMV viremia (58% versus 74%), fungal infection (4% versus 11%), and infection related death (4% versus 11%). At day 100, median CD4+ lymphocyte count was 229/mm^3^ for the MSD group and 190/mm^3^ for the haplo group. Despite these differences in immune recovery, TRM was similar between the groups [72]. Similarly, in our experience at the Moffitt Cancer Center, the recovery of total absolute CD4+ T cell count after haplo and MUD HCT with PTCy was significantly lower compared to MUD with calcineurin inhibitor (CNI)-based GVHD prophylaxis throughout 1 year of HCT [73]. In contrast, the total CD8+ T cell recovery was similar in all groups. A recent retrospective registry analysis by the Center for International Blood and Marrow Transplant Research compared haplo HCT with PTCy to matched donor HCT with PTCy to matched donor HCT with CNI for GVHD prophylaxis. Both PTCy groups had higher risks of CMV viremia, suggesting PTCy is an independent risk factor for CMV viremia regardless of donor type [74].

The TCD strategy results in the highest risks of infection with trials showing ~27% of treated patients dying of infection, a rate that is higher than all–cause transplant related mortality with many other platforms [70]. The most common infections reported were CMV and aspergillus. However, subsequent studies with this platform have shown potential for adoptive transfer of infection specific T-cells or regulatory T-cells to improve immune reconstitution and decrease infections [75,76].

Immune reconstitution of haplo HCT with the GIAC regimen has been prospectively compared to matched donor transplants [77]. In that study, survival outcomes were similar between the two groups, though CMV viremia was more prevalent in the haplo group: 50% versus 13% (*p* = 0.007). Compared to the matched donor group, the haplo group was noted to have decreased T-cell subsets and dendritic cells at day 90, with the most significant decreases observed in the CD4+ T-cells. Notably, B-cell recovery and monocyte recovery were similar between the two groups.

### 3.3. Graft-Versus-Tumor Effect after Haploidentical Transplant

Though HLA disparity is known to elicit a more potent GVT effect after transplant, rates of relapse after modern haplo donor HCT are similar or even higher than matched donor transplants in several reports [72,77,78]. The reasons for this are possibly related to the other components of the transplant platform such as intensity of the immune suppression associated with these regimens, as well as low intensity conditioning and/or bone marrow graft source often used in conjunction with haplo HCT with PTCy [79,80,81]. Additionally, relapse after haplo HCT often occurs, at least in part, through a unique mechanism through which the mismatched haplotype is eradicated from the tumor cells as a form of antigen escape [82,83,84]. This phenomenon, called “loss of heterozygosity,” occurs in up to 30% of relapses after haplo donor HCT but is rarely encountered in matched donor HCT [83,85,86]. As such, loss of heterozygosity is indirect evidence that the GVT effect in haplo donor HCT is driven by immune recognition of the HLA mismatch.

## 4. Umbilical Cord Blood Transplantation

No risk to the donor, rapid availability, less restrictive HLA-matching selection criteria, and low risk of chronic GVHD are well-recognized advantages of UCBT [87,88,89,90]. Thus, UCB as an alternative donor option has had large utilization in the past two decades offering curative allogeneic HCT to racial and ethnic minorities with various hematological malignancies. Conversely, the limitations of UCBT include delayed hematopoietic engraftment and immune reconstitution, leading to higher risks of infections and TRM after HCT [91,92,93,94,95]. The introduction of double UCBT and RIC further extended the access and made this alternative donor HCT option available to many adults with hematological malignancies [88,90,96,97,98]. However, slow immune reconstitution and higher frequency of infections still remain major obstacles to the successful use of UCB source [92,94].

### 4.1. Quantitative Immune Reconstitution after UCBT

We previously compared the pace of immune reconstitution after UCB (n = 89) and MSD peripheral blood (n = 68) allo HCT in patients receiving similar RIC (consisting of fludarabine (Flu), Cy and TBI) and GVHD prophylaxis [92]. Despite lower absolute numbers of total NK cells and individual NK cell subsets at day 28 after UCBT, their absolute numbers were significantly higher after UCBT compared to MSD HCT as early as day 60 after HCT. Similarly, despite lower absolute B cell count at day 28 the numbers of B cells were significantly higher at day 100 after UCBT compared to MSD HCT. Conversely, UCBT was associated with significantly slower recovery of CD8+ and CD4+ T cell subsets as compared to MSD HCT. While the numbers of most CD4+ T cell subsets (central memory, effector memory and regulatory) were lower after UCBT within only the first 100 days of HCT, the naïve CD4+ T cell count remained low throughout 6 months after HCT. For the CD8+ T cells subsets, the central memory CD8+ T-cell count was lower within the 100 days whereas the naïve and effector memory CD8+ T-cell counts remained significantly lower throughout 6 months after UCBT compared to MSD. The use of ATG had no significant impact on immune reconstitution in our analysis. We observed significantly higher frequency of viral infections within first 180 days and bacterial infections within first 60 days after UCBT compared to MSD HCT [92]. A similar pattern of more robust recovery of NK cells and B cells but slower recovery of T-cell immune subsets is reported after RIC UCBT as compared to MUD HCT [95].

Other studies also reported this distinct pattern of immune cell count recovery after either myeloablative or RIC UCBT [91,92,93,94,99].

### 4.2. Virus-Specific Immune Reconstitution after UCBT

Prior studies were largely focused on quantitative immune cell count recovery after UCBT. We recently compared virus-specific immune reconstitution after UCBT and MSD peripheral blood HCT in patients receiving the same RIC regimen with Flu, Cy, TBI and no ATG [100]. Interferon-gamma (IFN-γ) enzyme-linked immune absorbent spot assay (ELISpot), which was used to quantify the frequencies of IFN-γ-secreting peripheral blood mononuclear cell (PBMC), identified higher frequencies of CMV-specific PBMCs after HCT in CMV seropositive patients compared to CMV seronegative patients. However, the frequencies of CMV-reactive PBMCs in CMV seropositive recipients were significantly lower after UCBT compared to MSD HCT throughout the first 12 months after transplant. These findings suggest that higher rates of CMV reactivation/infection after UCBT are explained not only by delayed quantitative recovery of immune cells but also by slower recovery of CMV-specific immunity after UCBT compared to MSD HCT. The reconstitution of other virus-specific immunity (HHV6, EBV, BK and adenovirus) was not significantly different between the two donor types in our analysis [100]. Another prior study reported high rates of CMV, BK and adenovirus infections after myeloablative conditioning UCBT. The authors reported significant delay (up to 12 months) in recovery of virus–reactive PBMCs against CMV, EBV, BK, adenovirus, influenza and RSV antigens [94]. However, all patients in that study also received ATG in addition to myeloablative conditioning.

While CMV is the most frequently reported viral reactivation/infection after UCBT we also observed higher frequency of HHV6 reactivation/infection after UCBT with use of sirolimus instead of cyclosporine in combination with MMF as GVHD prophylaxis (51% vs. 20%; *p* < 0.01 by day +45) [98]. HHV6 reactivation is generally an earlier event after UCBT (median onset of 26 days) and can be associated with primary graft failure after RIC UCBT [98]. Introduction of antiviral prophylaxis with foscarnet from day +7 through neutrophil engraftment after UCBT delayed the time to HHV6 reactivation and resulted in higher neutrophil engraftment rates in our recent report [101].

## 5. Interventions to Mitigate Complications of Immune Deficiency after Allogeneic HCT

### 5.1. Prevention of Bacterial Infection

The first month of allogeneic transplant lends to a high risk of bacterial infections due to severe neutropenia and breakdown of mucosal barriers [8]. The primary pathways of entrance for these infections are translocation of oral/intestinal flora due to mucosal injury, or transmission of skin flora through indwelling catheters or skin breakdown [8]. Thus, both Gram-positive and Gram-negative bacteria are implicated, though Gram-negative bacteremia results in especially rapid clinical decline [102]. Because of this, antibacterial prophylaxis should be considered for all patients undergoing allogenic HCT [8]. Fluoroquinolones are the preferred agents based on prospective data and meta–analyses demonstrating improvements in infection related mortality and overall survival [103,104]. While ciprofloxacin is acceptable, levofloxacin is preferred in patients with poor dentition or high risk of mucosal injury given effectiveness against oral strep viridans [8]. In patients intolerant to fluoroquinolones, a recent retrospective study suggested similar efficacy with cefpodoxime as an alternative agent [105]. Antibacterial prophylaxis should be continued until neutropenia resolves, generally 2–3 weeks after the stem cell infusion. Notably, these general recommendations should be modified based on the local bacterial resistance patterns [106]. During the period of neutropenia, most centers also monitor closely in the inpatient setting. In the case of neutropenic fevers, broad spectrum antibiotics with pseudomonal coverage (e.g., piperacillin–tazobactam or cefepime) must be initiated within one hour of fever onset to decrease the risk of septic shock and mortality [107].

Though the risk of severe bacterial infection decreases after engraftment, patients with chronic GVHD are at high risk for encapsulated bacterial infections such as *Neisseria meningitides* or *Streptococcus pneumoniae* [108]. Therefore, prophylaxis with penicillin should be considered for patients on systemic glucocorticoid therapy for chronic GVHD.

### 5.2. Prevention of Fungal Infection

Fungal infections are common in the first month after HCT but the risk continues beyond engraftment, particularly in patients who develop GVHD [109]. The role for antifungal prophylaxis early after transplant is clear, with studies demonstrating significantly improved overall survival [110]. Candida and invasive molds, such as aspergillus, are most problematic, which would suggest prophylaxis against both is necessary. However, large, well conducted studies have demonstrated that prophylaxis against mold and candida with posaconazole or voriconazole does not reduce risks of invasive fungal infections nor improve overall survival compared to prophylaxis against candida with fluconazole [111,112]. Thus, candida prophylaxis with fluconazole through neutrophil engraftment is considered adequate for most allogeneic HCT patients. The echinocandins are also effective against candida species, with a broader spectrum than fluconazole, and may be substituted based on local resistance patters or side effect profiles [8,109]. However, mold coverage with voriconazole or posaconazole is implemented for patients with risk factors such as prolonged neutropenia or presence of lung nodules prior to transplant [8]. For patients who develop GVHD and require high doses of IST or prednisone (≥0.5 mg/kg), anti-fungal prophylaxis should be reinitiated [108]. In a randomized controlled phase III trial, posaconazole was superior to fluconazole in preventing invasive aspergillosis and reducing the mortality related to fungal infections in patients with GVHD who require systemic IST [113]. 

Pneumocystis jiroveci is a yeast-like fungus that causes pneumonia in patients with low CD4+ T-lymphocytes as well as those on IST or prednisone doses above 20 mg/kg/day [8,109]. PCP prophylaxis with trimethropim-sulfa, dapsone, atovaquone, or pentamidine is recommended for all allogeneic HCT patients for at least 6 months, and should be continued in the setting of ongoing IST [8].

### 5.3. Prevention of Viral Infection

Both herpes simplex virus (HSV) and varicella zoster virus (VZV) may be reactivated in immunocompromised patients after allogeneic HCT [8]. The antiviral acyclovir and valacyclovir are both similarly effective in reducing the risk of these viruses and are acceptable options for prophylaxis [114]. Continuing these drugs until the CD4+ T-cells are above 200/mm^3^ and IST has been discontinued is recommended to avoid rebound [8,115].

CMV disease remains a major cause of morbidity and mortality among allogeneic HCT patients [8]. After primary infection, the virus lies dormant in the myeloid cells. CD4+ and CD8+ T cells control the infection in healthy hosts. However, after allogeneic transplant, the period after myeloid recovery and preceding T-cell recovery (days 30–100) allows a window for CMV to reactivate [8]. Prevention of CMV infection starts with allogeneic donor selection. During donor selection, CMV serology should be tested in both the donor and the recipient. CMV seropositive patients with a CMV seronegative donor are at particularly high risk for CMV infection as the donor T-cells lack a CMV memory response to suppress the virus already present in the recipient’s body. Therefore, choosing a CMV seropositive donor reduces the risk of CMV reactivation and may improve overall survival [116]. Similarly, CMV negative patients benefit from a CMV negative donor to avoid transmission of CMV virus from the donor to the immunologically naïve host [116]. After HCT, weekly monitoring of CMV viral load by PCR should be checked and pre-emptive therapy with ganciclovir or valganciclovir or foscarnet should be started in asymptomatic patients with significant viremia in order to prevent CMV disease [8]. In high risk populations, including HLA-mismatched or haplo donors, UCBT, or ex vivo TCD, initiation of CMV prophylaxis is warranted. In a phase III trial, letermovir, compared to placebo, given for CMV prophylaxis in these high-risk groups resulted in significant reductions in CMV infection (37.5% versus 60.6%, *p* < 0.001) and all-cause mortality (10.2% versus 15.9%, *p* = 0.03) at 24 weeks post-HCT [45]. A recent study of haplo donor or HLA-mismatched unrelated donor HCT with PTCy similarly showed the risk of CMV reactivation was 22% with letermovir prophylaxis versus 69% with no prophylaxis (*p* < 0.001). Notably, CMV vaccines are currently being studied in phase III trials [117].

### 5.4. Immunizations after Allogeneic HCT

Following allogeneic HCT, humoral immunity is suppressed and antibody titers to previous vaccines decline [118,119,120]. This demonstrates that re-vaccination is necessary after transplant. Response to vaccines requires reconstitution of both T-cell and B-cell immunity and, specifically, naïve T-cells that are capable of mounting a memory response after exposure to a new antigen [16,17,29]. Thus, vaccination schedules commence approximately 3–12 months after transplant [17,30]. Specific practices may vary by institution both in terms of schedule and the choice of vaccinations administered. A typical approach is to begin the vaccination schedule with pneumococcal vaccine, followed by *Haemophilus influenzae*, tetanus/diphtheria/pertussis (DTaP), and hepatitis B. To avoid risk of infection, live vaccines including measles/mumps/rubella (MMR) or some shingles vaccines should not be given until patients are off of immune suppression and at least 2 years have passed since HCT [8,118,119,120]. Inactivated influenza vaccine should be administered yearly [8,109,121].

### 5.5. Interventions to Improve Immune Reconstitution

In addition to prophylaxis and vaccinations, strategies to improve immune reconstitution can potentially reduce the risks of infection and improve the GVT effect against relapse.

Modifications to GVHD prophylactic regimens can potentially improve the balance of immune reconstitution with risks of GVHD. Historically, IST regimens were continued for at least 90 days and tapered thereafter [4,5]. With the use of PTCy, clinical trials have shown that, depending on the conditioning and donor choice, IST can be discontinued prior to day 90 or even omitted completely without increasing risks of GVHD [122,123,124]. This has the potential to boost early immune reconstitution and, additionally, creates an optimal platform for adding post-HCT maintenance strategies to prevent relapse. For TCD grafts, novel graft manipulation techniques allow for selective depletion of alpha-beta T-cells and B-cells that cause GVHD while preserving transfer of the allogeneic gamma–delta T-cells and NK cells that are required for GVT activity and infection control [125]. A recent prospective trial of myeloablative haplo HCT with alpha-beta T-cell and B-cell depletion in acute leukemia resulted in no severe GVHD and overall survival of 75%, comparable to historic controls with matched donors [126].

Immune checkpoint inhibitors are drugs that activate the immune system to attack malignant cells [127]. The mechanism of action suggests potential utility in boosting the GVT effect if given in the post-HCT setting. A phase I trial of the CTLA-4 antibody ipilimumab for post-HCT relapse led to complete responses in 9% [128]. Responses correlated with CD8+ T-cell infiltration into the tumor, supporting the immune mediated mechanism of action. GVHD occurred in only 14%. The PD-1 inhibitor nivolumab has also been studied in the post–transplant setting, though fatal immune-related toxicities have limited the potential of this strategy [129]. Notably, patients treated with checkpoint inhibitors prior to HCT may also experience post-HCT immune effects including higher rates of GVHD [130]. However, studies of haplo HCT with PTCy after prior checkpoint inhibitor suggest that GVHD rates are acceptable and relapse rates may be lower than in patients who did not receive checkpoint inhibitors [131,132]. More data is needed to confirm these findings, but this suggests that haplo HCT with PTCy and checkpoint inhibition is a potential strategy to optimize GVT.

Transfer of exogenous cells to boost immunity may help boost post-HCT immune responses. Allogeneic donor lymphocyte infusions depleted of CD8+ T-cells have been shown to successfully treat relapse and reverse immune exhaustion with low rates of GVHD [51]. In haplo HCT, a phase I study of ex vivo-expanded, donor-derived NK cells infused with HCT resulted in no dose limiting toxicities and only 1 relapse among 13 patients treated [133]. The upcoming BMT CTN 1803 NK REALM phase II trial will evaluate the effectiveness of haplo NK cell infusion in reducing the risk of relapse after haplo HCT (NCT04395092). A number of studies have also explored exogenous cell transfer to manage infectious complications of allogeneic HCT [134]. Ex vivo virus specific T-cells can be generated from healthy donors with existing immunity to a specific viral pathogen and then infused into infected HCT patients. Virus specific T-cells have mostly been studied for treatment of CMV and EBV, with complete response rates as high as 75% [135,136,137]. However, virus specific T-cells are also in development for adenovirus, human herpes virus 6, and BK virus [135,138].

## 6. Conclusions

Allogeneic HCT is the only curative therapy for many high-risk hematologic malignancies. Advances in GVHD prevention have broadened the donor pool to include haplo related donors and UCBT. However, severe immune deficiency and subsequent infection and relapse remain primary drivers of post-transplant morbidity and mortality. The kinetics of immune reconstitution are useful for predicting the temporality of potential complications and implementing appropriate management strategies. Novel approaches in graft manipulation and adoptive cellular therapies are being studied to accelerate post-HCT immune recovery and improve outcomes.
